# Metronomic Chemotherapy in Triple-Negative Metastatic Breast Cancer: The Future Is Now?

**DOI:** 10.1155/2017/1683060

**Published:** 2017-12-03

**Authors:** M. E. Cazzaniga, L. Cortesi, A. Ferzi, L. Scaltriti, F. Cicchiello, M. Ciccarese, S. Della Torre, F. Villa, M. Giordano, C. Verusio, M. Nicolini, A. R. Gambaro, L. Zanlorenzi, E. Biraghi, E. Casini, L. Legramandi, E. Rulli

**Affiliations:** ^1^Research Unit Phase I Trials, ASST Monza, Monza, Italy; ^2^Oncology Unit, ASST Monza, Monza, Italy; ^3^Haematology and Oncology Unit, Azienda Ospedaliero-Universitaria Policlinico di Modena, Modena, Italy; ^4^Oncology Unit, ASST Ovest Milanese, Legnano, Italy; ^5^Oncology Day Hospital Unit, Ospedale Civile di Guastalla, Guastalla, Italy; ^6^Oncology Unit, Ospedale Vito Fazzi, Lecce, Italy; ^7^Oncology Unit, ASST Rhodense-Presidio di Garbagnate Milanese e Presidio di Rho, Garbagnate, Italy; ^8^Oncology Unit, ASST Lecco, Lecco, Italy; ^9^Oncology Unit, ASST Lariana, Como, Italy; ^10^Oncology Unit, ASST della Valle Olona, Saronno, Italy; ^11^Oncology Day Hospital Unit, Azienda USL Romagna, Cattolica, Italy; ^12^Oncology Unit, ASST Fatebenefratelli-Sacco, Milano, Italy; ^13^Oncology Unit, ASST della Valle Olona, Busto Arsizio, Italy; ^14^Oncology Unit, ASST Melegnano-Martesana, Gorgonzola, Italy; ^15^Methodology for Clinical Research Laboratory, IRCCS Istituto di Ricerche Farmacologiche Mario Negri, Milano, Italy

## Abstract

Triple-negative breast cancer (TNBC) shows a very bad prognosis, even in early stages of disease. Metronomic chemotherapy refers to the minimum biologically effective dose of a chemotherapy agent given as a continuous dosing regimen with no prolonged drug-free breaks that leads to antitumor activity. In the present article, we review preclinical and clinical data of metronomic administration of chemotherapy agents with or without biological agents in TNBC cell lines and patients, contextually reporting data from the VICTOR-2 study in the subgroup of patients with TNBC, in order to stimulate new ideas for the design of clinical trials in this subset of patients.

## 1. Introduction

Triple-negative breast cancer (TNBC), which accounts for 25% of the molecular subtypes, shows a very bad prognosis, even in early stages of disease [1]: after radical surgery, median time to relapse is approximately 18 months and from this point median overall survival is less than 24 months [2].

Different strategies have been studied to improve the prognosis of this subset of patients, and a lot of drugs are currently under evaluation. Despite the big efforts done to modify this clinical scenario, little or nothing has really changed in the last decades.

In elderly patients, the clinical scenario is, if possible, worse than expected: about 15% of breast cancers in older patients are of the triple-negative subtype [3] but only few of them receive adequate treatments, due to different reasons, mainly age-related factors such as comorbidities, deterioration of cognitive function, possible impairment in organ function, and the concomitant use of other drugs. All these factors must be carefully assessed to avoid or minimize toxicity risks.

In this context, low-dose metronomic chemotherapy (mCHT) might represent a promising therapeutic option for elderly TNBC women [3].

mCHT refers to the minimum biologically effective dose of a chemotherapeutic agent given as a continuous dosing regimen, with no prolonged drug-free breaks that leads to antitumor activity [4]. Till now, few data are available regarding the use of mCHT in TNBC patients [5–7]; most of these studies have been conducted in HER2-negative breast cancer patients and results in the TNBC subset are generally reported as subgroups analyses.

In the present article, we review preclinical and clinical data of metronomic administration of chemotherapy agents with or without biological agents in TNBC cell lines and patients, contextually reporting data from the VICTOR-2 study in the subgroup of patients with TNBC.

Available literature on the subject was identified by using PubMed with three different keywords [metronomic chemotherapy], [triple negative] or [TNBC] and [breast cancer], without any custom range for year of publication, journal, or article type, with the exclusion of results only published as abstract reports. This search resulted in 26 articles published between 2008 and 2017: five articles reporting reviews on this topic were excluded; another article was excluded due to the subject not strictly related to the object of the online search.

Here we report available literature data grouped by setting of treatment.

## 2. Preclinical Data

Di Desidero et al. [14] evaluated the potential therapeutic impact and molecular mechanisms of topotecan administered in continuous low-dose metronomic (LDM) manner, alone or in concurrent combination with pazopanib in a triple-negative, primary, and metastatic breast cancer orthotopic model; potential molecular mechanisms of efficacy were also studied, especially the impact of hypoxic conditions. The combination of metronomic topotecan and pazopanib significantly enhanced antitumor activity compared to monotherapy with either drug and prolonged survival, even in the advanced metastatic survival setting, with a marked decrease in tumor vascularity, proliferative index, and the induction of apoptosis. Significant changes in tumor angiogenesis, cancer cell proliferation, apoptosis, HIF1*α* levels, HIF-1 target genes, and ABCG2 were found both* in vitro *and in tumor tissue. The authors concluded that the combination of metronomic topotecan and pazopanib warrants further investigations being a potential treatment option for this poor prognosis group of breast cancer patients.

## 3. Clinical Data

### 3.1. Neo/Adjuvant Setting

Different authors reported trials which have included metronomic chemotherapy in the regimens studied.

The largest randomized Phase III trial by Colleoni et al. [5] randomized 724 TNBC patients as part of a larger study (IBCSG 22-00) to receive the metronomic combination of CM (CTX 50 mg/day orally continuously for 1 year and MTX 2,5 m/twice a day orally, days 1 and 2 of every week for 1 year) or placebo after a standard adjuvant treatment. The reduction in DFS events was not statistically significant for maintenance CM versus no maintenance (HR = 0.84; 95% CI 0.66–1.06); however, in the TNBC/N+ group (*n* = 340), the estimated 5-year DFS was 72.5% for the CM maintenance group versus 64.6% for the non-CM group (HR = 0.72; 95% CI 0.49–1.05). Patients with TNBC and node-positive disease had a nonstatistically significant reduced HR (*n* = 340; HR, 0.72; 95% CI, 0.49 to 1.05).

The right selection of patients and the right choice of drugs, both with regard to doses as well as schedules, are crucial factors for determining the success or the failure of metronomic chemotherapy in the adjuvant setting: Pruneri et al. [6], by analyzing the prognostic and predictive value of tumor-infiltrating lymphocytes (TILs) in the TNBC cohort of the IBCSG trial 22-00, identified a subgroup of tumors, the so-called lymphocyte-predominant breast cancer (LPBC), for which metronomic CM confers a greater, even not statistically significant, clinical benefit.

Nasr et al. [7] investigated the role of oral methotrexate plus Cyclophosphamide given in a metronomic schedule for 1 year after finishing the adjuvant treatment for patients with TNBC in an attempt to prolong their disease free interval. The primary study objectives were to compare the disease free survival (DFS) and OS for TNBC patients after adjuvant chemotherapy, who underwent maintenance metronomic chemotherapy versus no maintenance therapy. The secondary end point was toxicity. Patients were randomly assigned to receive FEC-100 [FEC-100 was given in the form of 5-fluorouracil 500 mg/m2, epirubicin 100 mg/m2, and Cyclophosphamide 500 mg/m2 (day 1)] for 3 cycles, followed by Docetaxel 80 mg/m2 plus Carboplatin AUC 5 for 3 cycles then metronomic chemotherapy or the same FEC part followed by Docetaxel 100 mg/m2 for 3 cycles without any maintenance metronomic chemotherapy. Metronomic maintenance chemotherapy consisted of oral Cyclophosphamide (50 mg PO daily) and methotrexate (2.5 mg PO BID on days 1 and 2 of each week).

The median DFS for the two groups were 28 and 24 months, respectively. The median OS for the two groups were 37 and 29 months, respectively. Additionally, during the follow-up period, the overall distant metastasis recurrence rates for the two cohorts were 26% and 37%, respectively.

The authors concluded that extended adjuvant metronomic chemotherapy achieved significant improvement in the survival and was well-tolerated.

In another study, Masuda et al. [8] studied the effects of preoperative metronomic combination of paclitaxel, Cyclophosphamide, and capecitabine (mPCX) followed by 5-fluorouracil (FU), epirubicin, and Cyclophosphamide (FEC) as preoperative chemotherapy in 40 TNBC patients. The primary end point of the study was the pathological complete response (pCR) rate. The pCR rate was 47.5% (19/40) whereas the clinical response rate was 90.0%. The authors reported a high incidence of severe adverse events, namely, neutropenia (35%), leukopenia (25%), and hand-foot syndrome (8%): these data are very different from the those reported by the vast majority of trials involving metronomic chemotherapy.

Different studies have also explored the role of metronomic chemotherapy in the adjuvant setting of treatment, mainly as prolonged therapy after a “standard” regimen.

Considering that there is no universally accepted standard chemotherapy regimen for adjuvant treatment of TNBC and classical regimens are currently reasonable choices, different authors tested alternative strategies with the aim of improving relapse free survival.

Shawky and Galal [9] investigated the tolerability of 1-year of metronomic capecitabine (650 mg/m2, twice every day) preceded by standard adjuvant therapy and overall survival in 19 patients with operable TNBC. The authors concluded that one year of capecitabine metronomic therapy preceded by standard adjuvant chemotherapy is active and well-tolerated in TNBC patients previously treated with standard adjuvant chemotherapy. With all the limits given by the small sample size and the single-arm design, the findings coming from this paper open important scenario for the future. In another Phase II trial, Alagizy et al. [10] evaluated the tolerability and efficacy of metronomic capecitabine as extended adjuvant treatment for women with triple-negative breast cancer. Forty-one patients received capecitabine 500 mg per os twice daily continuously for six months after finishing six cycles of adjuvant FEC100 ± postoperative radiotherapy. Even if this trial was not sufficiently powered to address the question regarding the role of angiogenesis bloc at the source by using metronomic chemotherapy, it was pioneer for subsequent trials investigating the same question, such as the BEATRICE trial and the IBCSG 22-00 one, published some years later.

It is our opinion that the use of metronomic chemotherapy, without strong preclinical data indicating which drugs should be used, how long, and at which doses, should not be adopted in the adjuvant setting.

Results of metronomic CHT in (neo)adjuvant setting are reported in Table 1.

### 3.2. Metastatic Setting

The literature only occasionally reports trials conducted with metronomic chemotherapy exclusively in TNBC patients being the majority of them case reports or analyses of subgroups of patients enrolled as part of trials conducted in HER2-negative patients.

Yoshimoto et al. [11] treated 45 patients, of whom only 9 were TNBC, with capecitabine 828 mg/m(2) twice daily with Cyclophosphamide 33 mg/m(2) twice daily, days 1–14 every 3 weeks. The primary endpoint was overall response rate (ORR). Secondary endpoints included progression-free survival (PFS), overall survival (OS), and safety. The median follow-up was 18.1 months. The authors reported an ORR of 44.4% and a clinical benefit rate (CBR) of 57.8% in the TNBC population. Median PFS was 12.3 months in the whole population and 10.7 months in triple-negative disease. Grade 3 adverse events comprised leukopenia (26%), neutropenia (16%), and decreased hemoglobin (2%). There was no grade 3 hand-foot syndrome. The authors concluded that oral XC is an effective first- or second-line therapy for MBC, demonstrating high activity in both luminal A and triple-negative disease with few severe side effects, but the small sample size of TNBC group strongly affected transposition of these results.

Wang et al. [12] explored the combination of chemotherapy with immunotherapy, followed by maintenance metronomic Cyclophosphamide as a potential alternative option for the treatment of patients with metastatic TNBC. Results reported were strongly influenced by the induction phase of the trial and the authors do not report data for the metronomic maintenance part of the trial.

Kummar et al. [13] conducted a Phase II randomized trial in order to assess the role of PARP inhibition in the treatment of TNBC patients; Veliparib, a small molecule PARP inhibitor, was administered with the Cyclophosphamide 50 mg once daily and compared with Cyclophosphamide same dose alone in patients with refractory TNBC. The authors demonstrated that the addition of Veliparib to Cyclophosphamide did not improve the response rate (CR + PR) over Cyclophosphamide treatment alone.

Kontani et al. [15] analyzed 80 patients with MBC who received chemotherapy in the metastatic setting, comparing clinic-pathological factors and clinical outcomes between 52 patients who received metronomic regimens and 28 patients who received other cytotoxic regimens. The median time-to-treatment failure (TTF) and overall survival (OS) were significantly longer in the metronomic group compared with those in the nonmetronomic group; however, none of the 18 patients who responded to the regimen had triple-negative (TN) cancer. TTF and OS were significantly longer in patients with non-TN cancer compared with those in patients with TN cancer in the metronomic group (TTF, 16 versus 7 months, *P* = 0.0014; OS, 108 versus 20 months, *P* = 0.000007, resp.). The authors concluded that metronomic chemotherapy could be a viable option for luminal-type MBC but at the same time an alternative treatment is required for TN cancer.

Results of the main studies regarding the role of metronomic CHT in the metastatic setting are reported in Table 2.

**Figure 1:**
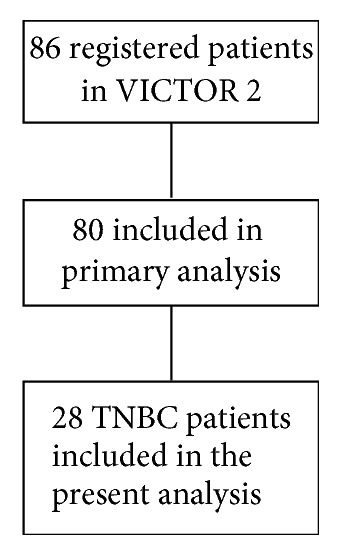
Consort diagram VICTOR 2 trial.

The VICTOR-2 is a Phase II, single-arm study evaluating the efficacy and safety of the metronomic combination of Vinorelbine (VNR), 40 mg three times per week, and capecitabine (CAPE) 500 mg three times per day, continuously, in 80 HER2-negative breast cancer patients. Twenty-eight out of 80 (35%) were TNBC (Figure 1). Patients and methods, as well as results regarding the whole population, have been reported elsewhere [16]. Median age in the TNBC group was 69 years (47–85), 23 patients (82.1%) had visceral involvement at the time of enrolment, and only 4 (14.8%) had less than 2 sites of disease. Fifteen patients received mCHT as second or further line of treatment. The clinical benefit rate (CBR) was 35.7% (95% CI: 18.6–55.9) and the median duration of CB was 11.3 months. Disease control rate (DCR; CR + PR + SD) was 53.7% and median duration of disease control was 7.4 months. Progression-Free Survival (PFS) was 4.7 months.  Table 3 summarizes efficacy results. Severe toxicity did not exceed 8% and was mainly hematologic.

To our knowledge, these are the first prospective data ever published regarding the activity of mCHT in a population of metastatic TNBC patients.

Different drugs and regimens have been tested in TNBC patients, with the aim of disease control and survival prolongation.

Platinum salts, including carboplatin and cisplatin, lead to DNA cross-link strand breaks, which may be especially important in cells that are deficient in homologous recombination repair mechanisms such as* BRCA1/2*-associated tumors and TNBCs. The Phase III TNT (Triple-Negative Breast Cancer Trial) study compared carboplatin area under the curve (AUC) 6 every 3 weeks with docetaxel 100 mg/m2 every 3 weeks as first-line treatment for advanced stage disease. In the overall population, at a median follow-up of 11 months, PFS was 4.5 and 3.1 months, respectively, not so different from what we observed in our trial, while taking into account the differences in terms of study design between the two trials.

Our results do not significantly differ from those obtained by other authors with Eribulin, even in first-line setting: the ORR was 16.7%, the CBR was 25.0%, and median PFS was 3.4 months in 12 patients with TNBC treated with Eribulin in a Phase II study [17].

Few data are available regarding the use of nab-paclitaxel, a novel formulation of exclusively TNBC patients: in a Phase II study [18] first-line treatment with nab-paclitaxel, carboplatin, and Bevacizumab was associated with an ORR of 85%, a CBR of 94%, and a median PFS of 9.2 months. The study enrolled 34 patients and reported grade 3/4 adverse events in 53% and 18% of the patients (neutropenia and thrombocytopenia, resp.). In the VICTOR-2 study grade 3-4 leucopenia was observed in 7 patients (8.8%) and grade 3-4 thrombocytopenia in 2 patients (2.5%).

Our results suggest that metronomic combination of VNR and CAPE could represent a further treatment option for TNBC patients and for this reason could be considered in special populations, such as the elderly ones.

### 3.3. Toxicity

The evaluation of toxicity clearly related to metronomic chemotherapy is difficult to be done due to the fact that most trials describe general toxicity and not those specifically related to metronomic regimens, thus putting together those related to the nonmetronomic part of the regimen studies. The second issue is that, in trials specifically addressed to evaluate metronomic chemotherapy, there is often nondistinction between toxicities reported in TNBC patients and non-TRBC ones.

However, there could be no reason to split toxicities according to biological subtype, considering that specifically toxicities are mainly related to the regimen although to the type of disease.

We briefly summarize hereafter the toxicities described in trials specifically addressed to metronomic chemotherapy.

In the study by Colleoni et al. [5], the authors reported that, of 473 patients who received at least one CM maintenance dose (including two patients assigned to no CM), 64 (14%) experienced a grade 3 or 4 treatment-related adverse event; elevated serum transaminases were the most frequently reported (7%), followed by leukopenia (2%), but they did not distinguish toxicities occurring in TNBC patients from those observed in non-TNBC ones.

Nasr et al. [7] detailed the toxicity occurring in the metronomic part of the treatment, reporting grade 3 neutropenia in 2.8% of the patients, grade 3 anemia in 1.5%, and vomiting in 11% of the patients, respectively.

Shawky and Galal [9] reported that treatment-related adverse events were manageable with only 1 patient (5.3%) suffering from grade 3/4 hand-foot syndrome and another 1 patient (5.3%) suffering from grade 3 diarrhea. No grade 3/4 hematologic toxicity was recorded.

Yoshimoto et al. [11] reported grade 3 adverse events in their patients treated with capecitabine 828 mg/m^2^ twice daily with Cyclophosphamide 33 mg/m^2^ twice daily, days 1–14 every 3 weeks, mainly leukopenia (26%), neutropenia (16%), and decreased hemoglobin (2%). There was no grade 3 hand-foot syndrome.

However, considering the doses used and the schedule, this regimen cannot be considered, according to the widely accepted definition of metronomic chemotherapy, a truly metronomic regimen, and this could explain the high rates of toxicity observed in these patients, which are almost different from those reported by the vast majority of metronomic trials, even in non-TNBC patients [5, 16].

## 4. Conclusion

At the moment, few data, mainly obtained by Phase II trials, are available regarding the use of metronomic chemotherapy in TNBC patients; however, others are on the way, exploring different settings.

An international, randomized Phase II study (VICTOR-3) is currently ongoing to investigate the role, as maintenance therapy, of metronomic VNR, either single agent or in combination with metronomic CTX, in TNBC patients, after an induction chemotherapy with standard-dose regimens.

The CAMELLIA (NCT01917279) trial was designed to explore the efficacy and safety of metronomic CTX versus intermittent standard-dose CTX as maintenance therapy after first-line therapy with CTX plus docetaxel in HER2-negative metastatic breast cancer.

Even if the future is probably not now for the routine use of metronomic chemotherapy in TNBC patients, some promising results are ready to consider this regimen in particular subgroups, such as the elderly ones, for whom few therapeutic options exist.

## Figures and Tables

**Table 1:** Summary of data in the (neo)adjuvant setting.

Author (year)	Setting/type of trial	Drug(s)	Number of patients	Results
Pruneri et al. [6]	Adj/Phase III	CTX 50 mg/day orally continuously for 1 year and MTX 2.5 mg/twice a day orally, days 1 and 2 of every week for 1 year versus no maintenance chemotherapy	647	BCFI risk reduction: 13% DFS risk reduction: 11% DFRI risk reduction: 16% OS risk reduction: 17% (for every 10% increase of TILs)

Nasr et al. [7]	Adj/Phase II	*Group 1*	158 (whole population) 78	mDFS 28 versus 24 months mOS 37 versus 29 Distant mets recurrence rate 26% versus 37%
		FEC-100 × 3 cycles		
		Docetaxel 80 mg/m^2^ + Carboplatin AUC 5 *Followed by*		
		CTX 50 mg/day + MTX 2,5 mg bid, days 1 and 2 every week		

Masuda et al. [8]	(Neo)adj/Phase II	mPTX 80 mg/m^2^ days 1, 8, and 15		pCR 47.5% cORR 90%
		CTX 50 mg/day		
		CAPE 1200 mg/m^2^, daily	40	
		*Followed by*		
		FEC100		

Shawky and Galal [9]	Adj/Phase II	mCAPE 650 mg/m^2^, twice every day, after standard adjuvant chemotherapy for 1 year	19	2 ys-DFS rate 88.8% 3 ys-DFS rate 82.05%

Alagizy et al. [10]	Adj/Phase II	CAPE 500 mg twice daily continuously for 6 months after finishing six cycles of adjuvant FEC100	41	Mean DFS 42.4 months

EPI = epirubicin; CDDP = cisplatin; 5FU = 5-fluorouracil; PTX = paclitaxel; CTX = Cyclophosphamide; CAPE = capecitabine; mCAPE = metronomic capecitabine; FEC100 = 5FU + EPI 100 mg/m^2^ + CTX; MTX = methotrexate; cCR = clinical complete response; cPR = clinical partial response; pCR = pathologic complete response; DFS = disease free survival; BCFI = breast cancer free interval; DFRI = distant recurrence free interval; OS = overall survival.

**Table 2:** Summary of data in the metastatic setting.

Author (year)	Line of treatment Type of trial	Drug(s)	Number of patients evaluable for end points	Results
Yoshimoto et al. [11]	1st-2nd line Phase II	CAPE 828 mg/m^2^ twice daily + CTX 33 mg/m^2^ twice daily, dd 1 → 14, every 21 days	9	ORR 44.4% CBR 57.8% Median PFS 10.7 months

Wang et al. [12]	2nd line or further (maintenance) Phase II	CTX 50 mg daily (after CTX 3 g/m^2^ for the preparation of CD34+ and CTX 3 g/m^2^, thiotepa 150 mg/m^2^, and CBDCA AUC = 6, every 28 dd for 2 courses)	23	NA

Kummar et al. [13]	2nd line or further Phase II	CTX 50 mg/day versus CTX 50 mg/day + Veliparib 60 mg once daily throughout a 21-day cycle	39	ORR 5.6% versus 9.5% (NS)

ORR = overall response rate; CBR = clinical benefit rate; PFS = progression-free survival; CBDCA = carboplatin.

**Table 3:** Efficacy results of VICTOR trial in the TNBC population.

Variable	Overall
	*N* = 28
Clinical benefit rate (CR + PR + SD ≥ 24 weeks) *n*/*N* (%)	10/28 (35.7)
[95% CI]	[18.6–55.9]
Kaplan-Meier estimate of median duration of clinical benefit (months)	11.3
Disease control rate (CR + PR + SD) *n*/*N*	15/28 (53.6%)
Kaplan-Meier estimate of median duration of response in disease control (months)	7.4
Kaplan-Meier estimate of median PFS (months)	4.7
